# Progress of natural sesquiterpenoids in the treatment of hepatocellular carcinoma

**DOI:** 10.3389/fonc.2024.1445222

**Published:** 2024-07-16

**Authors:** Xiaodong Wang, Fancheng Meng, Jingxin Mao

**Affiliations:** ^1^ Department of Medical Technology, Chongqing Medical and Pharmaceutical College, Chongqing, China; ^2^ College of Pharmaceutical Sciences, Southwest University, Chongqing, China

**Keywords:** hepatocellular carcinoma, natural sesquiterpenoid, bioactivity, resources, pharmacological mechanism

## Abstract

Hepatocellular carcinoma is one of the common malignant tumors of digestive tract, which seriously threatens the life of patients due to its high incidence rate, strong invasion, metastasis, and prognosis. At present, the main methods for preventing and treating HCC include medication, surgery, and intervention, but patients frequently encounter with specific adverse reactions or side effects. Many Traditional Chinese medicine can improve liver function, reduce liver cancer recurrence and have unique advantages in the treatment of HCC because of their acting mode of multi-target, multi-pathway, multi-component, and multi-level. Sesquiterpenoids, a class of natural products which are widely present in nature and exhibit good anti-tumor activity, and many of them possess good potential for the treatment of HCC. This article reviewed the anti-tumor activities, natural resources, pharmacological mechanism of natural sesquiterpenoids against HCC, providing the theoretical basis for the prevention and treatment of HCC and a comprehensive understanding of their potential for development of new clinical drugs.

## 1 Introduction

Cancer is one of the major causes of death in the world, and its incidence rate rises sharply with age. In 2022, 412 thousand patients died from liver cancer among 3.2 million cancer-caused deaths in China (1). The data is 32 thousand deaths in more than 640 thousand cancer-caused deaths in US. The incidence and death rates caused by liver cancer are increasing rapidly in recent years and liver cancer is now the fastest-increasing cause of cancer death (2–4). Liver cancer had been the fifth most common cause of cancer deaths in men, and the eighth most common cause of cancer deaths in women in US (5). Hepatocellular carcinoma (HCC) is the most common type of liver cancer in adults (6). Hepatitis B and C, alcoholism, and nonalcoholic steatohepatitis, cirrhosis, and other factors exacerbate the morbidity of HCC (7, 8). However, HCC was frequently discovered in its advanced stage and lack of effective therapeutic methods. Unsatisfactory therapeutic efficacy, the recurrence of cancer, adverse effects and the occurrence of drug resistance make it critical to develop alternative strategies for the treatment of HCC.

Discovering active compounds from natural products and subsequently developing new drugs for cancer treatment is a feasible strategy (9). 113 of 136 approved small molecule anticancer drugs either are natural products or inspired by natural products (10). Among the herbal medicines and the natural products with antitumor effects on hepatocellular carcinoma (11–14), sesquiterpenoids, which are widely distributed in various angiosperms, a few gymnosperms and bryophytes, displayed significant anti-cancer activities and possessed high potential against liver cancer (15). Structurally, the anti-HCC sesquiterpenoids can be classified into artemisinin and its derivatives, bisabolane-type, *β*-caryophyllene and *β*-caryophyllene oxide, elemane-type, eudesmane-type, germacrane-type, guaiane-type, pseudoguaiane-type, santhane-type, their dimers, etc. Different sesquiterpenoids exhibited anti-HCC activity through modulation of different signaling pathways. This review summarized the advances in anti-HCC sesquiterpenoids and their mechanism to provide the current status of natural sesquiterpenoids as promising anti-liver cancer treatments.

## 2 Pathways involved in anti-HCC effect by sesquiterpenoids

**Figure 1:**
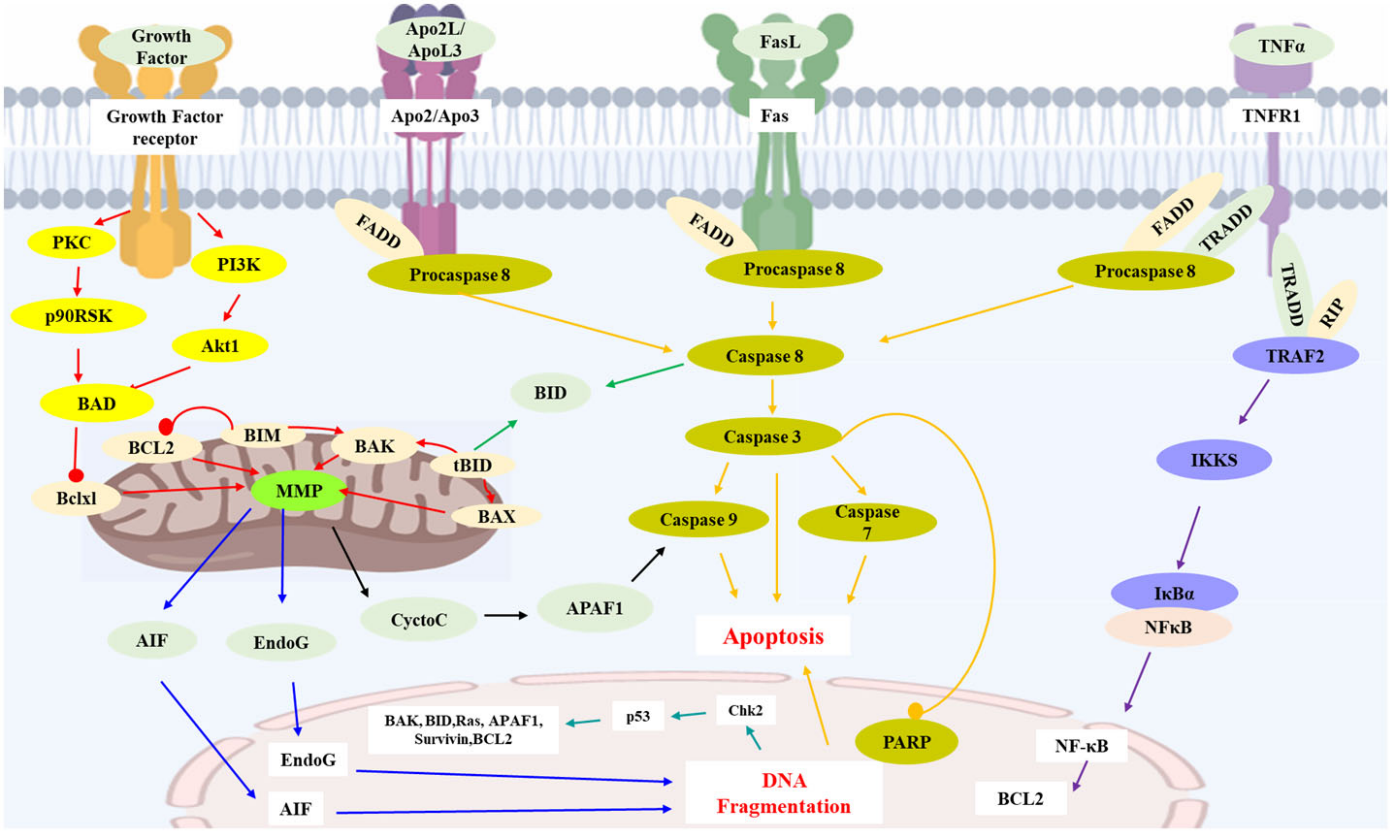
Key apoptosis pathways involved in sesquiterpenoids-treated hepatocellular carcinoma. Caspase family proteins in the cytoplasm are the main enzymes that promote cell apoptosis and can be classified into two categories: initiator caspases (caspase-8, 9, etc.), and executioner caspases (caspase-3, *etc.*). In mitochondrial pathway, protein of Bcl-2 family (Bcl-2, Bcl-xl, Bax, Bak, Bid, etc.) insert into the outer membrane pores of mitochondria in the form of oligomers and cause Ca2+ influx, resulting in loss of MMP and release of Cyt C from mitochondria into cytoplasm. Release of Cyt C could activate caspases and execute apoptosis. Apaf-1, AIF and Endo G are also the proapoptotic proteins involved in mitochondrial mediated apoptosis. In death receptor pathway, death receptors (TNFR-1, Fas, etc.) would activate caspase-8 and trigger apoptosis. TNFR-1 could also induce apoptosis via IKK/NF-κB axis. P53 protein is an anti-cancer transcription factor. Wild type p53 protein induces cell apoptosis by preventing DNA replication in the G1 phase of the cell cycle, and turning off transcription of the Bcl-2 gene. AIF: Apoptosis inducing factor; APAF1: Apoptotic protease activating factor 1; Apo2L: Apo2 ligand; ApoL3: Apo3 ligand; BCL2: B cell lymphoma 2; BAD: Bcl2-antagonist of cell death; BAK: Bcl2 antagonist killer 1; BAX: Bcl2 associated X-protein; Bclxl: Bcl2 related protein long isoform; BID: BH3 interacting death domain; tBID: Truncated BID; BIM: Bcl2 interacting protein; Chk2: Check point kinase 2; Cycto C: Cytochrome C; EndoG: Endonuclease G; FasL: Fas ligand; FADD: Fas-associated death domain; IKKS: I-Kappa B kinases; MMP: Mitochondrial membrane potential; NFκB: Nuclear transcription factor-κB; p90RSK: p90 ribosomal-S6 kinases; PARP: Poly ADP-ribose polymerase; PKC: Protein kinase C; PI3K: Phosphoinositide 3-kinase; RIP: Receptor-interacting protein 1; TNFα: Tumor necrosis factor α; TNFR1: Tumor necrosis factor receptor 1; TRADD: Tumor necrosis factor receptor 1-associated death domain; TRAF2: TNF receptor associated factor 2.

By summarizing the literature, 48 sesquiterpenoids were reported their anti-HCC effect. The anti-HCC effect and underlying mechanism of natural sesquiterpenoids were listed in **Table 1**. **Figure 1** summarized the related pathways and molecules in the apoptosis induced by sesquiterpenoids. Apoptosis refers to the active and physiological process of cell death under physiological and pathological conditions (75–77), which is regulated by apoptosis-related genes and hence known as programmed cell death (PCD), the main form of cell death. Apoptotic cell death generally includes three steps. The first phase is an instruction of death issued to target cell. Followed by an execution phase occurs, which is characterized by a series of stereotypic morphological changes in the cell structure, including nuclear agglutination, cellular shrinkage, formation of membrane vesicles, loss of microvilli, degradation of chromosomal DNA, and formation of DNA fragments in nucleosome length units. Phagocytosis phases occurs with apoptotic bodies digested and engulfed by surrounding phagocytic cells. Mitochondrial pathway (or intrinsic pathway), death receptor pathway (or extrinsic pathway), and endoplasmic reticulum pathways are the main signaling pathways that mediate apoptosis (78). They interact with each other and jointly regulate the process of apoptosis.

**Table 1:** Anti-HCC effect and mechanism of natural sesquiterpenoids.

No.	Compounds	Source	Activity^a^	Mechanism	Rf.
1	Artemisinin	*artemisia annua*	HepG2, SMMC-7721, Male athymic BALB/c nu/nu mice bearing HepG2 orthotopic xenografts (50, 100 mg/kg/d, 4 weeks)	Inhibit invasion and metastasis	(16)
2	Dihydroartemisinin	derivative of artemisinin	HepG2 (42.76), PLC/PRF/5, Hep3B (109.76), SK-Hep-1, Huh-7 (79.74), L02, mice bearing HepG2 xenografts (20 mg/kg, five times a week)	induce G2/M cell cycle arrest, apoptosis, autophagy-dependent caspase-independent cell death, DAPK1-BECLIN1 pathway	(17–21)
3	Artesunate	derivative of artemisinin	HepG2, BWTG3, SK-Hep-1, SM-7721, Wild-type 129S2/SvPasCrl male mice received weekly intraperitoneal saline (30 mg/kg/day, 5 weeks), Athymic BALB/c nu/nu mice injected subcutaneously with SK-hep1 cells (100 mg/kg/day, 4 weeks)	induce apoptosis by inhibiting STAT-3 and PI3K/AKT/mTOR pathway	(22–25)
4	Xanthorrhizol	*Curcuma xanthorrhiza*	HepG2 (4.17)	inducing apoptosis through causing DNA fragmentation	(26, 27)
5	*α*-Bisabolol	*Matricaria chamomilla*, *Vanillosmopsis arborea*, *Nectandra megapotamicav*	HepG2	induce apoptosis via cleavage of PARP and activation of caspases-3, -8 and -9, and mitochondrial pathway	(28)
6	1*β*, 8-Diangeloyloxy-2*β*-acetoxy-4*α*-chloro-11-methoxy-3*β*, 10-dihydroxybisabola-7 (14)-ene	*Cremanthodium discoideum*	SMMC-7721	induce cell-cycle arrest at G1 phase	(29)
7	1*β*, 8-Diangeloyloxy-2*β*-acetoxy-4*α*-chloro-3*β*-hydroxy-10, 11-O, O-isopropylidenebis-aboia-7 (14)-ene	*Cremanthodium discoideum*	SMMC-7721	induce cell-cycle arrest at G1 phase	(29)
8	*β*-Caryophyllene	plants of *Eugenia*, hops, *Psidium*, *Origanum*, *Betula*, *Liquidambar*, *Bidens*, and *Abies*	HepG2, Alexander, PCL/PRF/5	sensitize HCC cells to sorafenib and doxorubicin	(30, 31)
9	*β*-Caryophyllene oxide	plants of *Eugenia*, hops, *Psidium*, *Origanum*, *Betula*, *Liquidambar*, *Bidens*, and *Abies*	HepG2, Alexander, PCL/PRF/5	sensitize HCC cells to sorafenib and doxorubicin	(30, 32, 33)
10	*β*-Elemene	*Curcuma aromatica*	HepG2	induce apoptosis and G2/M phase cell cycle arrest	(34)
11	–	derivative of *β*-elemene	H22 liver cancer xenograft mouse (30, 60 mg/kg, 3 weeks)	suppress the growth of H22 liver cancer xenograft	(35)
12	Igalan	*Inula helenium*	HepG2	induce quinone reductase, inactivate GSK3*β* and activate AKT to induce Nrf2 activation, inhibit NF‐κB activation	(36)
13	Alantolactone	*Inula* genus	HepG2 (33), Bel-7402, SMMC-7721	mitochondria-mediated apoptosis, ROS mediated AKT pathway	(37–39)
14	Isoalantolactone	*Inula* genus	Hep3B	induce apoptosis by inducing ROS-dependent activation of JNK signaling pathway	(40)
15	2*α*,5*α*-Dihydroxy-11*αH*-eudesma-4 (15)-en-12,8*β*-olide	*Carpesium abrotanoides*	HepG2 (9.83)	reduce the expression of JAK2 and STAT3	(41)
16	Telekin	*Carpesium abrotanoides*	HepG2 (2.95)	activate mitochondria-mediated apoptosis	(41, 42)
17	Oxoeudesm-11 (13)-eno-12,8*α*-lactone	*Carpesium abrotanoides*	HepG2 (4.15)	reduce the expression of JAK2 and STAT3	(41)
18	Cryptomeridiol	*Magnolia officinalis*	HepG2, Huh-7, Hep3B	aggravate the pre-activated UPR and activated the silenced orphan nuclear receptor Nur77	(43)
19	Santamarine	–	HepG2 (70)	modulation of mitochondrial signaling pathway (ROS↑, TrxR↓, GSH↓, MMP↓, modulation of Bcl-2 family proteins, Cytochrome C release, etc.)	(44)
20	*α*-Eudesmol	*Guatteria friesiana*	HepG2 (9.71)	induce apoptosis by inducing MMP loss and increasing caspase-3 activation	(45)
21	*β*-Eudesmol	*Guatteria friesiana*	HepG2 (24.57)	induce apoptosis by inducing MMP loss and increasing caspase-3 activation	(45)
22	*γ*-Eudesmol	*Guatteria friesiana*	HepG2 (9.50)	induce apoptosis by inducing MMP loss and increasing caspase-3 activation	(45)
23	Germacrone	*Curcuma* genus	HepG2, HepG2 xenograft BALB/c Female nude mice (5, 10, 15 or 20 mg/kg, 3 weeks)	increase ROS production, promote cleavage of caspase 3 and gasdermin E, promote pyroptosis	(46)
24	Furanodiene	*Curcuma* genus	HepG2 (80.9); SMMC-7721 (67.2)	induce cell arrest at G2/M and apoptosis by activating mitochondrial signaling pathway	(47)
25	Parthenolide	*Tanacetum parthenium*	Hep3B, PLC/PRF/5, SK-Hep-1, MHCC97-H, H22-bearing wild-type BALB/c mice (5, 10 mg/kg/d, 15 days)	Induce depletion of glutathione and ROS generation, activate mitochondrial signaling pathway, caspases-7, -8, and -9, induce cell cycle arrest at G2/M phase, triggering apoptosis	(48)
26	Costunolide	*Dolomiaea souliei*, *Aucklandia lappa*	HepG2 (18.09); HA22T/VGH (4.7)	Mitosis arrest	(49, 50)
27	Onopordopicrin	*Shangwua* genus	HepG2, L02	targeting inhibit TrxR and induce oxidative stress-mediated tumor cell apoptosis	(51)
28	Tagitinin C	*Tithonia diversifolia*	HepG2 (5.7), HepG2 xenograft female BALB/c nude mice (15 μg/mouse/day intraperitoneally, 3-4 days)		(52)
29	Deoxyelephantopin	*Elephantopus scaber*	HepG2	induce apoptosis by promoting ROS generation, glutathione depletion, loss of MMP, expression of Bcl-2 proteins, cytochrome c release, and PARP cleavage	(53)
30	Elephantopinolide J	*Elephantopus scaber*	HepG2 (2.83); Hep3B (1.98)	induce autophagy, cell arrest at G2/M phase and apoptosis by enhancing the ROS production, decreasing MMP	(54)
31	Achillin	*Artemisia ludovisiana*	Hep3B/PTX	chemosensitizer of paclitaxel, induce G2/M phase cell cycle arrest and promote apoptosis	(55)
32	Dehydrocostuslactone	*Dolomiaea souliei*, *Aucklandia lappa*	HepG2 (16.7); PLC/PRF/5 (18.8)	induces ER stress and mitochondrial apoptosis	(56)
33	Hemistepsin A	*Hemistepta lyrata*	HepG2, Huh-7, Hep3B, Huh7-bearing BALB/c-nu mice (5, 10 mg/kg/3days, 9 days)	induce G0/G1-phase arrest, cellular senescence and mitochondrial-related apoptosis, downregulation of STAT3	(57, 58)
34	Mecheliolide	Aucklandiae Radix	HepG2, Hepa 1–6	act as an inhibitor of TrxR and induce ICD-associated damage-associated molecular patterns	(59)
35	Bigelovin	*Inula hupehensis*	HepG2/STAT3 cells (3.37), HepG2 and SMMC-7721, L02, HepG2 xenograft male athymic BALB/c nude mice (5, 10. 20 mg/kg/2days, 21 days)	regulate JAK2/STAT3 signaling and induce apoptosis	(60, 61)
36	Brevilin A	*Centipeda minima*	HepG2 (13.1), SMMC-7221 (17.7)	inactivation of STAT3/Snail and Wnt/*β*-catenin signaling pathways	(62)
37	Britannin	*Inula aucheriana*	Bel-7402 (2.702); HepG2 (6.006), Huh-7 (27.86), SMMC-7721 (28.92), L02, HepG2 xenograft male BALB/c nu/nu nude mice (7.5, 15, 30 mg/kg/d, i.p., 21 days)	induce apoptosis and autophagy by activating AMPK and NF-κB signaling pathway	(63, 64)
38	Tomentosin		HepG2, Huh-7	induced DNA fragmentation, cell cycle arrest at the SubG1 and G2/M stage and apoptosis	(65)
39	Xanthatin	*Xanthium strumarium*	HepG2 (4.48), Bel-7402 (6.07), SMMC-7721 (5.10), L02	arrest cell cycle at the G2/M phase at 10 μM, and at the S phase at 40 μM, induce apoptosis by activating ERS	(66)
40	1*β*-Hydroxyl-5*α*-chloro-8-epi-xanthatin	*Xanthium sibiricum*	SK-Hep-1 (1.567), HepG2 (1.630), SMMC-7721 (2.401), Huh-7(2.123)	induce apoptosis via ROS-mediated ERK/p38 MAPK activation and JAK2/STAT3 inhibition	(67)
41	Gossypol	cotton seeds	HepG2 (72h 6.30, 96h 4.35), Hep3B (72h 6.87, 96h 5.83)	pan-histone deacetylase inhibitor, induce apoptosis via caspase activation	(68)
42	Shizukaol D	*Chloranthus serratus*	SMMC-7721 (8.82), SK-Hep-1 (9.25), HepG2 (>50)	induced apoptosis by repressing Wnt/β-catenin signaling	(69)
43	Artemeriopodin G7	*Artemisia eriopoda*	Huh-7 (18.3), SK-Hep-1 (19.0), THLE-2 (32.0)	induce cell cycle arrest in G2/M phase and apoptosis by inhibiting AKT/STAT signaling pathway	(70)
44	(–)-Agelasidine A	*Agelas nakamurai*	HepG2 (135.5), Hep3B (70.1), primary mouse hepatocyte cells (213.9)	Induce apoptosis via activation of mitochondrial signaling pathway and ER stress	(71)
45	Cordycepol C	*Cordyceps ophioglossoides*	SMMC-7721 (42.1)	induce caspase-independent apoptosis by causing PARP-1 cleavage and MMP loss	(72)
46	Epiroridin acid	endophytic fungus *Myrothecium roridum* derived from *Pogostemon cablin*	LX-2 (0.477), HepG2	activate caspase-9 and 3, up-regulated Bax level, down-regulated bcl-2 level, and reduced MMP	(73)
47	Mytoxin B	endophytic fungus *Myrothecium roridum* derived from *Pogostemon cablin*	LX-2 (0.004), HepG2	activate caspase-9 and 3, up-regulated Bax level, down-regulated bcl-2 level, and reduced MMP	(73)
48	Melleolide	*Armillaria mellea*	HepG2 (4.95), L02 (16.05)	increase expressions of cleaved caspase 3, 8, 9, Bax and Ki67, and induce G2/M phase cell cycle arrest	(74)

a Cell lines (IC_50_ μM) or/and animal model (dosage, duration).

### 2.1 Mitochondrial pathway

Mitochondria, the regulatory centers of cell apoptosis, medicates apoptosis by disrupting the antioxidant capacity, hindering the production of ATP through disrupting the electron transfer chain and the oxidative phosphorylation process, and regulating mitochondrial pathway (79).

Cytochrome C (Cyt C) is an important regulatory factor for mitochondrial apoptosis encoded by nuclear genes, and generally exists between outer and inner membranes of mitochondria. Under normal physiological conditions, Cyt C serves as a carrier that can transfer electrons in the mitochondrial respiratory chain, establish mitochondrial transmembrane potential, and produce ATP (80, 81). When apoptosis signaling is initiated, Cyt C is released from mitochondria into cytoplasm and plays an essential role in apoptotic cell death (82).

The Bcl-2 protein family consists of approximately 20 homologues of important anti- and pro-apoptotic regulators of PCD, including Bcl-2, Bcl-xl, Bal-xs, Bad, Bax, Bak, Bik, and Bid (83, 84). Among them, Bcl-2 and Bcl-xl exert anti-apoptotic activity. Bal-xs, Bad, Bax, Bak, Bik, and Bid have the effect of promoting apoptosis (85). The Bcl-2 proteins generally exist in the form of dimer, whose functions are modulated by its subgroup proteins of Bax and Bcl-xl proteins (86). Bcl-2 and Bcl-xL form heterodimers with Bax and Bak to maintain the integrity of the mitochondrial outer membrane and prevent mitochondrial apoptosis (87). When stimulated by DNA damage, growth factor deficiency, or other factors, the expressions of Bax and BH3 proteins increase and bind to Bcl-2 and Bcl-xL, hence cause the formation of Bax/Bak oligomers which could insert into the outer membrane pores of mitochondria and form ion channels (88). Then, Ca^2+^ influx results in changes in mitochondrial osmotic pressure and loss of mitochondrial membrane potential.

Caspase family proteins which distribute in the cytoplasm, belong to cysteine protease systems with similar structures and are the main enzymes that promote cell apoptosis (89). Caspase proteins can be classified into two categories: initiator caspases such as caspase-8, -9, and -10, and executioner caspases such as caspase-3, -6, and -7 (90). Caspase-9 is the initiator caspase of intrinsic pathway of apoptosis, while caspase-8 and caspase-10 are the initiators of extrinsic pathway of apoptosis. Caspase-3 is a key enzyme in the execution of apoptosis whereas its activation leads to the induction of apoptosis (91). Currently, caspase-3 and caspase-9 were considered to be most related to the mitochondrial apoptotic pathway.

When Cyt C was released from mitochondria to the cytoplasm due to decreased mitochondrial membrane potential (MMP), inactive caspases are activated and execute apoptosis (92). Cyt C in cytoplasm combines with apoptotic protease activating factor 1 (Apaf-1, a tumor suppressor gene (93, 94)) to form an apoptotic complex, and activates the precursor of Caspase-9, thereby activating Caspase-3 and Caspase-7, triggering a caspase cascade reaction and inducing cell apoptosis (95). Moreover, the deficiency or dysfunction of Cyt C can cause abnormal respiratory chain function, hinder ATP generation, and lead to cell apoptosis.

In addition to Cyt C, apoptosis inducing factor (AIF), second mitochondrial activator of caspases (SMAC), serine protease HtrA serine peptidase 2 (Omi/HtrA2), Endo G are also the proapoptotic proteins involved in mitochondrial mediated apoptosis (96–99). During the process of apoptosis, these proteins are released from the mitochondrial membrane gap into the cytoplasm. SMAC and Omi/HtrA2 can bind to inhibitors of apoptosis (IAP) and counteract their inhibition of IAPs to caspase 3 and 9, inducing caspase-dependent apoptosis. While, AIF and Endo G can be translocated to the nucleus, causing chromatin condensation and large-scale DNA fragmentation, inducing caspase-independent apoptosis.

Protein tyrosine phosphatase (PTP), embedded between the inner and outer membranes of mitochondria, mediates the formation of mitochondrial permeability transition pore (MPTP), which can selectively transport molecules less than 1.5 Kd (100). When challenging by high osmotic pressure, the channel opens and causes apoptosis. Both Cyt C and PTP pathways can cause a decrease in MMP. Therefore, a decrease in MMP is a characteristic marker of apoptosis.

### 2.2 Death receptor pathway

Death receptors (DR) belong to the tumor necrosis factor receptor TNFR gene superfamily, including TNFR-1, Fas (CD95), DR3, TRAIL-R1 (DR4), and TRAIL-R2 (DR5) (101, 102). Death inducing signaling complex (DISC) are constructed by binding of proapoptotic FasL and TRAIL with Fas (CD95), TRAIL-R1 (DR4), and TRAIL-R2 (DR5), respectively (103). DISC contains adapter molecules FADD and procaspase-8. Procaspase-8 would be induced to activated caspase-8 by self-cleavage and initiate downstream caspase cascade reactions (104). Activated caspase-8 can cleave pro-apoptotic protein Bid to yield a p15 C-terminal truncated fragment (tBid), which would be translocated to mitochondria and binds to the mitochondrial membrane to release pro-apoptotic substances and triggering an apoptotic response (105).

In TNFR-1 mediated apoptosis, the trimeric TNFR recruit adapter protein TRADD, which binds to FADD, RIP, and TRAF-2 through its DD to form the cytosolic complexes (106). Combination of TRADD with FADD could lead to oligomerization of procaspase-8, triggering caspase cascade reaction and apoptosis. While, combination of TRADD with RIP will activate TRAF-2 to form the TRADD-RIP-TRAF-2 complex, which further activates NF-κB induced kinase (NIK) through phosphorylation. NIK could activate IκB kinase (IKK), thereby leading the phosphorylation and activation of IκB, and degradation and release of NF-κB. Translocation of NF-κB to the nucleus plays an important role in activating transcription of some anti-apoptotic genes, such as Bcl2, c-IAP1, and then exerting anti-apoptotic effects by inhibiting the activation of caspase-8 and other pathways. Therefore, TNFR may be involved in apoptosis and anti-apoptosis, two completely opposite signaling pathways. After activation of TNFR pathway, actors such as the strength of the signaling levels of both parties determine the direction of cell development.

### 2.3 Endoplasmic reticulum pathway

Endoplasmic reticulum is responsible for protein synthesis, processing, and modification in cells. In cases of viral infection, calcium homeostasis disorders, etc., the accumulation and aggregation of unfolded proteins lead to severe endoplasmic reticulum stress response (ER stress) (107). ER stress could mediate apoptosis by different pathways. Under unfolded protein response (UPR) conditions, Inositol-requiring enzyme 1 (IRE1) can activate XBP-1 mRNA, induce transcription and translation of ER molecular chaperones gene, and alleviate ER stress (108–110). IRE1 can also combine with TRAF2 to increase ER stress through kinase activity and activate ASK and JNK protein kinases in the presence of ASK1 to initiate the apoptotic cascade.

C/EBP homologous protein (CHOP) maintains low levels of expression in the cytoplasm under normal circumstances (111). When ER stress occurs, the dissociation of Bip/GRp78 and endoplasmic reticulum transmembrane proteins PERK, ATF6, etc. can activate CHOP. CHOP further induces the expressions of apoptotic proteins (such as GADD34, ERO1, DR5), which can directly activate GADD34 and promote the biosynthesis of downstream ER proteins. Overexpression of CHOP can lead to the production of reactive oxygen species (ROS), trigger ER stress, and mediate apoptosis (112). Meanwhile, the excessive expression of CHOP can also cause a decrease in Bcl-2 expression, leading to transport of Bax from the cytoplasm to mitochondria and initiating the mitochondrial apoptosis.

### 2.4 P53 pathway

P53 protein is a transcription factor with anti-cancer effects. Wild type p53 protein induces cell apoptosis by preventing DNA replication in the G1 phase of the cell cycle, and turning off transcription of the Bcl-2 gene (113). Apaf-1 and caspase-9 are the downstream components of P53 in tumor cell apoptosis, involved in mitochondrial mediated apoptosis pathways. In the presence of dATP, Apaf-1 could activate caspase-3.

Additionally, Ca^2+^ also plays an important role in both the mitochondrial and endoplasmic reticulum apoptotic pathways (114, 115). Ca^2+^ concentration is crucial in maintaining mitochondrial membrane permeability and endoplasmic reticulum stability. An increase in mitochondrial Ca^2+^ concentration leads to the production of ROS, which in turn releases Cyt C and induces apoptosis. Changes of the endoplasmic reticulum state can also trigger ER stress, activate the JNK pathway, stimulate Bax activation, and induces apoptosis.

## 3 Anti-HCC mechanism of active sesquiterpenoids

### 3.1 Artemisinin and its derivatives

**Figure 2:**
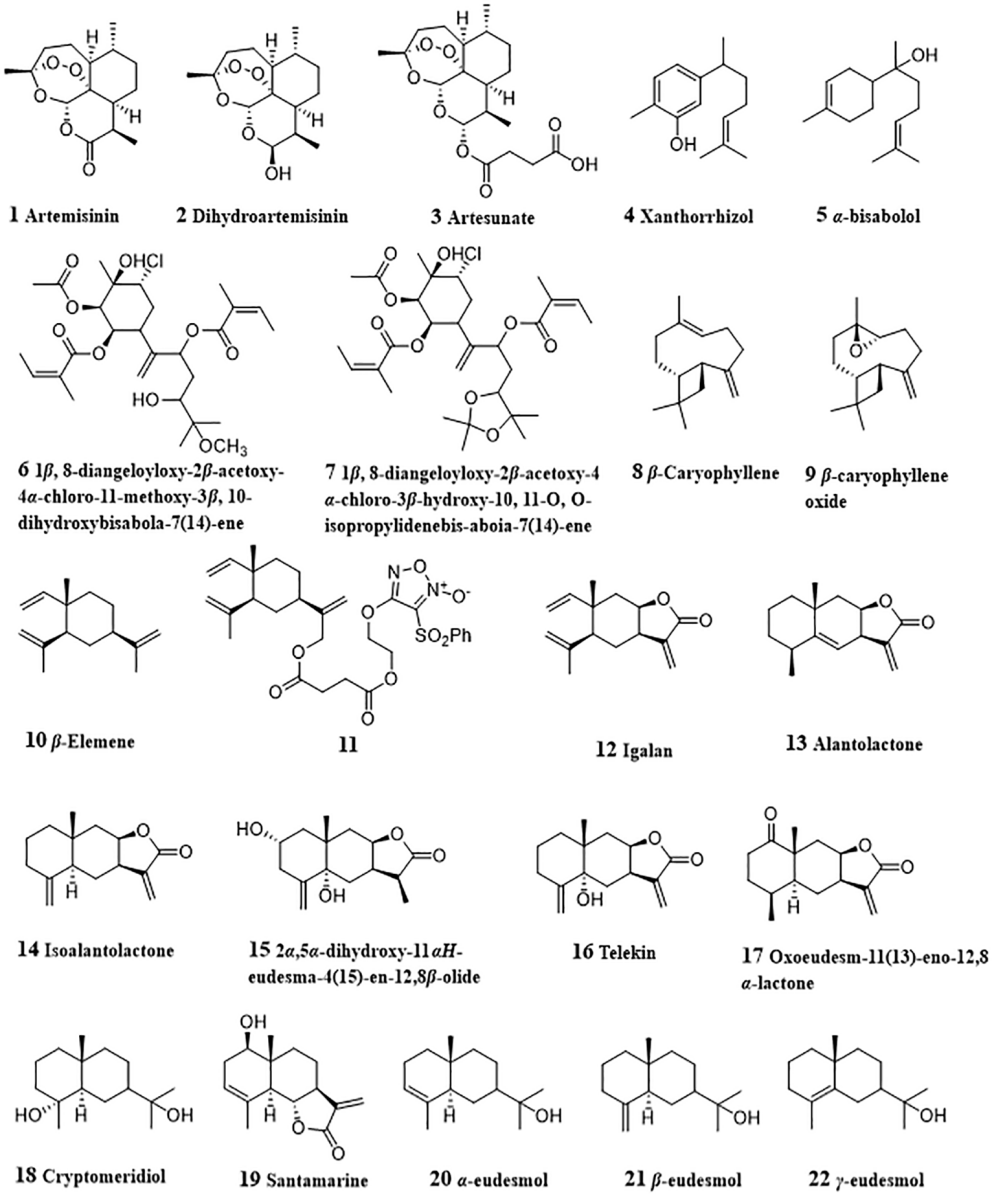
Structures of natural anti-HCC sesquiterpenoids (1 – 22).

Artemisinin (**1**, **Figure 2**) is a famous natural sesquiterpenoid obtained from *artemisia annua* for its treatment effect against malaria and the fact that due to the study on artemisinin, YouYou Tu won the 2015 Nobel Prize in Physiology or Medicine (116). Emerging evidence indicates that artemisinin has potent anti-tumor effects against various types of cancer cells (117). Tan et al. investigated the effects of artemisinin on the invasion and metastasis of hepatocellular carcinoma G2 (HepG2) and SMMC-7721 cells *in vitro* and *in vivo* and found that artemisinin could suppress p38, ERK1/2 activation, alter the balance between matrix metalloprotease 2 and TIMP2, and increase adhesion of tumor cells via Cdc42 activation (16).

Dihydroartemisinin (**2**) is the reduced derivative of artemisinin and exhibits HCC cell growth inhibitory activities *in vitro* and *in vivo* by inducing G2/M cell cycle arrest and apoptosis (17). Dihydroartemisinin-induced G2/M arrest was related with the induction of p21 and the inhibition of cyclin B and CDC25C. Dihydroartemisinin treatment led to the mitochondrial membrane depolarization, release of cytochrome c, activation of caspase 9 and caspase 3, and DNA fragmentation in HepG2, PLC/PRF/5 and Hep3B cell lines, thereby inducing apoptosis of HCC cell lines. Facilitated release of Bak from Mcl-1/Bak suppressor complex due to dihydroartemisinin-induced Mcl-1 degradation was also associated with dihydroartemisinin-induced apoptosis. Qin et al. (18) reported that increased Bim expression medicated by dihydroartemisinin played a part in regulating the Bak/Mcl-1 rheostat and apoptosis induction in HCC cells. In SKHep-1 cells, dihydroartemisinin could activate caspase 3, caspase 8, and caspase 9, cleave poly(ADP-ribose) polymerase (PARP), down-regulate Sp1 protein expression and nuclear localization, and decrease the phosphorylation of ERK, p38 and JNK (19). In 2018, Thongchot et al. studied the *in vitro* anti-cholangiocarcinoma activity of dihydroartemisinin against MMNK-1, KKU-023, KKU-100, KKU-213, KKU-452 cell lines (20). Dihydroartemisinin (12.5 - 50 *μ*M) displayed toxic effect in all cells. Further study on the underlying mechanisms showed that dihydroartemisinin induced both apoptosis and autophagy-dependent caspase-independent cell death, while being slightly toxic to immortalized cholangiocytes. The expression of DAPK1 and the interaction of BECLIN1 with PI3KC3 were promoted, and the interaction of BECLIN1 with BCL-2 was reduced by dihydroartemisinin. All these findings indicated that dihydroartemisinin-induced apoptosis was related with the Sp1 pathway, mitogen−activated protein kinase (MAPK) pathway and DAPK1-BECLIN1 pathway. Xiao et al. designed a biomimetic nanomedicine of dihydroartemisinin, which targets HepG2 cancer cells both *in vitro* and *in vivo* (21). This nanomedicine was made by loading dihydroartemisinin in a ZIF-8 core doped with ferrous ion and then encapsulating the ZIF-8 core with a cancer cell membrane-derived shell. This nanomedicine had better anti-HCC activity due to the high drug loading efficiency and controllable drug release.

Artesunate (**3**), a reduced artemisinin succinate monoester, has dose-dependently and time-dependently inhibiting effect on the viability of HepG2 and BWTG3 cells (22). Artesunate could induce HCC apoptosis due to its specifically targeting inhibition of STAT-3 (23). Artesunate interfered with STAT-3 dimerization and suppression of both constitutive and IL-6 inducible STAT-3 *in vitro*, and then modulated STAT-3 dependent procaspase-3, Bcl-xl and survivin. In another study, artesunate was found to induce apoptosis of HCC cells by its inhibitory effect on PI3K/AKT/mTOR pathway (24). Interestingly, it was found that artesunate could promote sensitivity to sorafenib and has a synergistic anti-tumor effect in HCC cell lines. Co-treated of artesunate with 2.77 mM sorafenib was equal to 5.23 mM sorafenib alone for 50% inhibition of SK. This synergistic effect was achieved via improved tumor cell apoptosis by the enhanced inhibition in both MAPK and PI3K/AKT/mTOR pathways. Similar results were also discovered by Li et al. (25). When co-treated with sorafenib (0.5 - 2 *μ*M), the induction of death in Huh7, SNU-449, and SNU-182 cells was facilitated, and HCC tumor growth *in vivo* was also inhibited. Further study on the molecular mechanisms indicated that impaired mitochondrial functions by combined treatment of artesunate with sorafenib increased the generation of mitochondrial-derived ROS and repressed ATP production, thereby inducing ROS-dependent cell death. Meanwhile, artemisinin and sorafenib could synergistically induce HCC ferroptosis via increased lipid peroxidation, lysosomal activation, and ferritin degradation.

### 3.2 Bisabolane-type sesquiterpenoids

Xanthorrhizol (**4**) was derived from *Curcuma xanthorrhiza* and had anti-cancer activities against liver, lung, breast, cervical, colon cancers, and other types of cancer by alleviating angiogenesis and metastasis, activating apoptosis and inducing cell cycle arrest (26). In HepG2 cells, proteolytic cleavage of PARP and DFF45/ICAD were promoted by xanthorrhizol, which indicated the DNA fragmentation. Meanwhile, the expressions of anti-apoptotic Bcl-2 and Bcl-xl were also reduced. All these results suggested xanthorrhizol as a potent anti-HCC agent by inducing apoptosis (27). *α*-bisabolol (**5**) is rich in many edible and ornamental plants, such as *Matricaria chamomilla*, *Vanillosmopsis arborea*, *Nectandra megapotamicav* (118) and displayed various therapeutic and biological properties, including anti-oxidative, anti-inflammatory, anti-cancers, and others (119). It was reported that *α*-bisabolol induced apoptosis via cleavage of PARP and activation of caspases-3, -8 and -9 in HepG2 cells. Mitochondrial pathway was involved in *α*-bisabolol-induced apoptosis, which was evidenced by increased release of cytochrome c and expression of Bak, and decreased expression of Bcl-2, Bax and Bid. Increased the expression of p53, NF-*κ*B and Fas revealed their participation in the apoptosis induced by *α*-bisabolol (28). Two bisabolane-type sesquiterpenes, from *Cremanthodium discoideum*, namely 1*β*, 8-diangeloyloxy-2*β*-acetoxy-4*α*-chloro-11-methoxy-3*β*, 10-dihydroxybisabola-7 (14)-ene (**6**) and 1*β*, 8-diangeloyloxy-2*β*-acetoxy-4*α*-chloro-3*β*-hydroxy-10, 11-O, O-isopropylidenebis-aboia-7 (14)-ene (**7**), could decrease *α*-foetoprotein content, reduce γ-glutamyl transferase activity, inhibit tyrosine-α-ketoglutarate transaminase in SMMC-7721 cells and induce cell-cycle arrest at G1 phase (29).

### 3.3 *β*-Caryophyllene and *β*-caryophyllene oxide

*β*-Caryophyllene (**8**, CP) and *β*-caryophyllene oxide (**9**, CPO) are two sesquiterpenoids widely distributed in plants of *Eugenia*, hops, *Psidium*, *Origanum*, *Betula*, *Liquidambar*, *Bidens*, and *Abies* (120). CP and CPO could sensitize HCC cells to sorafenib and doxorubicin. Isobolographic analysis indicated that better cytotoxic effect of sorafenib in combination with CP and CPO were achieved by a synergistic interaction. CP and CPO reduced the intracellular accumulation of rhodamine 123 and calcein acetoxymethyl ester (calcein-AM) in HepG2 cells. Further study showed that reduction of calcein-AM efflux was due to the inhibition of the activities and expressions of ATP-binding cassette (ABC) transporters, including P-glycoprotein (P-gp or MDR1) and multidrug resistance protein 1/2, suggesting that STAT3/ABC-transporters axis played an important role in sorafenib chemosensitization by sorafenib with CP or CPO (30). Similar inhibition effect on efflux activities of MDR1, MRP1 and MRP2 by CPO were also observed in HCC Alexander/R cells (32). CP and CPO could potentiate the efficacy doxorubicin in a lower dose level and reduce the occurrences of severe side effects. CP and CPO induced the intracellular accumulation of doxorubicin and rhodamine 123 in HepG2 cells. At the same time, compared with administration with doxorubicin alone, doxorubicin combined with CP or CPO lowered the expression of P-gp. This indicated that CP and CPO enhanced doxorubicin-sensitivity of HepG2 cells through decreasing doxorubicin efflux by P-gp (33). In cholangiocarcinoma Mz-ChA-1 cells, CP also showed doxorubicin chemosensitization effects by suppressing STAT3 phosphorylation and inducing ROS-increase and upregulating of GSH defenses (31). All above findings suggested that CP and CPO had the potential to be developed as chemosensitizing agents for the treatment of HCC and cholangiocarcinoma.

### 3.4 Elemane-type sesquiterpenoids

*β*-Elemene (**10**) is a natural sesquiterpene from *Curcuma aromatica* Salisb with anti-tumor effect. *β*-elemene inhibited the growth of HepG2 cells in a time- and dose- dependent manner, and induced apoptosis and G2/M phase cell cycle arrest of HepG2 cells (34). *β*-elemene-induced apoptosis was related with the up-regulated expressions of Fas and FasL. Due to the good anti-cancer potential of *β*-elemene, Chen et al. (35) synthesized a series of novel furoxan-based NO-donating *β*-elemene hybrids to improve its anticancer efficacy. Among them, compound **11** displayed significantly suppressing activity on the growth of H22 liver cancer xenograft with a tumor inhibitory ratio of 64.8%. Igalan (**12**) is a sesquiterpene lactone existing in *Inula helenium* L. and could induce quinone reductase (a marker for cancer prevention) activity in HepG2 cells (36). Igalan also inactivated GSK3*β* and activated AKT to induce nuclear factor erythroid 2‐related factor 2 (Nrf2) activation in HepG2 cells. The nuclear accumulation of Nrf2 led its target genes, HO‐1 and NQO1 increased. Meanwhile, igalan inhibited the tumor necrosis factor‐α (TNF‐α)‐induced nuclear factor‐κB activation and suppressed the expression of its target genes (TNF‐α, interleukin (IL)‐6, and IL‐8). These results indicated that igalan had the potential to be developed as a detoxifying agent.

### 3.5 Eudesmane-type sesquiterpenoids

Alantolactone (**13**) is an active eudesmane-type sesquiterpenoid lactone with diverse pharmacological effects originated from plants of *Inula* genus (Compositae) (121). In various types of cancer cell lines, alantolactone displayed anti-proliferation activities (122), such as A549, Hela, MCF-7, MDA-MB-231, HepG2, Bel-7402 and SMMC-7721 cell lines. In HepG2 cells, alantolactone could induce HepG2 cells apoptosis by blocking cell cycle arrest in G2/M phase (37). Further study showed that alantolactone treatment could up-regulate Bax, Bak, down-regulate Bcl-2, decrease MMP, thereby promoting cytochrome c release, activating caspase-9, caspase-8, caspase-3, and Bid cleavage, and leading mitochondria-mediated apoptosis. In another study by Khan et al. (38), apoptosis of HepG2 cells induced by alantolactone was also associated with GSH depletion, ROS generation and inhibition of STAT3 activation. Reduced the intracellular GSH resulted in oxidative stress and consequently led reduced phosphorylated STAT3 level and MMP dissipation. Activated mitochondrial apoptotic pathway led increased Bax/Bcl-2 ratio and caspase-3 activation, which promoted the process of apoptosis. ROS mediated AKT pathway may also was involved in alantolactone-induced apoptosis (39). In HepG2 cells treated with alantolactone, the accumulation of ROS and attenuated p-AKT expression revealed that alantolactone could induce apoptosis of HepG2 cells through ROS/AKT pathway. Alantolactone also significantly reduced the expressions of PINK1 and Parkin, inhibited the mitophagy in HepG2 cells, which promoted the apoptosis.

Isoalantolactone (**14**), an isomer of alantolactone from plants of *Inula* genus, also displayed multiple bioactivities including anti-HCC activity (123, 124). In Hep3B cells treated with isoalantolactone, increased expressions of caspase-3, caspase-8, and caspase9, and degradation of PARP were observed. Mitochondrial pathway-related proteins, Bax and tBid were increased, while Bcl-2 was decreased accompanying with the concentration-dependent loss of MMP. Meanwhile, increased ROS levels when treatment of Hep3B cells with isoalantolactone could be suppressed by pretreatment with NAC, suggesting the relation between ROS production induced by isoalantolactone and its cytotoxicity. The levels of p-ERK, p-JNK, and p38 related to MAPK signaling pathway were also increased when administrated with isoalantolactone and JNK inhibitor (SP600125) could block the expression of DRs and the degradation of PARP, and reverse isoalantolactone-induced apoptosis. Moreover, co-treatment with NAC could reduce isoalantolactone-induced JNK activation. All these results indicated that isoalantolactone induced Hep3B cells apoptosis by inducing ROS-dependent activation of JNK signaling pathway (40).

From *Carpesium abrotanoides*, Shen et al. isolated three eudesmane-type sesquiterpene lactone, 2*α*,5*α*-dihydroxy-11*αH*-eudesma-4 (15)-en-12,8*β*-olide (**15**), telekin (**16**), and oxoeudesm-11 (13)-eno-12,8*α*-lactone (**17**). All these three compounds exhibited anti-HCC activities through reducing the expression of JAK2 and STAT3 and inhibiting the expression of *p*-JAK2 and *p*-STAT3 levels in the HepG2 cells (41). Telekin induced apoptosis due to its down-regulation of Apaf-1 and Bcl-2 expression, up-regulation of Bax expression, promoting release of cytochrome C, activation of caspase-9 and caspase-3, thereby activating the mitochondria-mediated apoptotic pathway (42).

Cryptomeridiol (**18**), a natural sesquiterpenoid from *Magnolia officinalis*, aggravated the pre-activated UPR and activated the silenced orphan nuclear receptor Nur77 in HCC cells (43). Mechanistically, Nur77 was translocated to the mitochondria by sensing IRE1α-ASK1-JNK signaling, and led to MMP loss. At the same time, the aggravation of ER stress and mitochondrial dysfunction induced by cryptomeridiol increased cytotoxic ROS production. Santamarine (**19**) dose-dependently induced apoptosis of HepG2 cells with IC_50_ about 70 μM (44). This apoptosis inductive effect was achieved via modulation of mitochondrial signaling pathway (up-regulation of ROS generation, down-regulation of thioredoxin reductase (TrxR) activity, glutathione (GSH) depletion, MMP dissipation, modulation of Bcl-2 family proteins, cytochrome c release, caspases-9, -8 and -3 activation and PARP cleavage). Moreover, santamarine decreased the phosphorylation of IkB-α and SATA3, blocking the translocation of NF-кB into nucleus and activation of STAT3, which assisted in apoptosis induction. Bomfim et al. (45) investigated the antiproliferative activity effect of three eudesmol isomers (*α*-, *β*-, *γ*-eudesmol, **20** - **22**) and the results showed that they displayed cytotoxic activity against HepG2 cells with IC50 values ranging from 42.72 to 110.49 μM. Further study revealed that three eudesmol isomers induced apoptosis of HepG2 cells by inducing MMP loss and increasing caspase-3 activation.

### 3.6 Germacrane-type sesquiterpenoids

**Figure 3:**
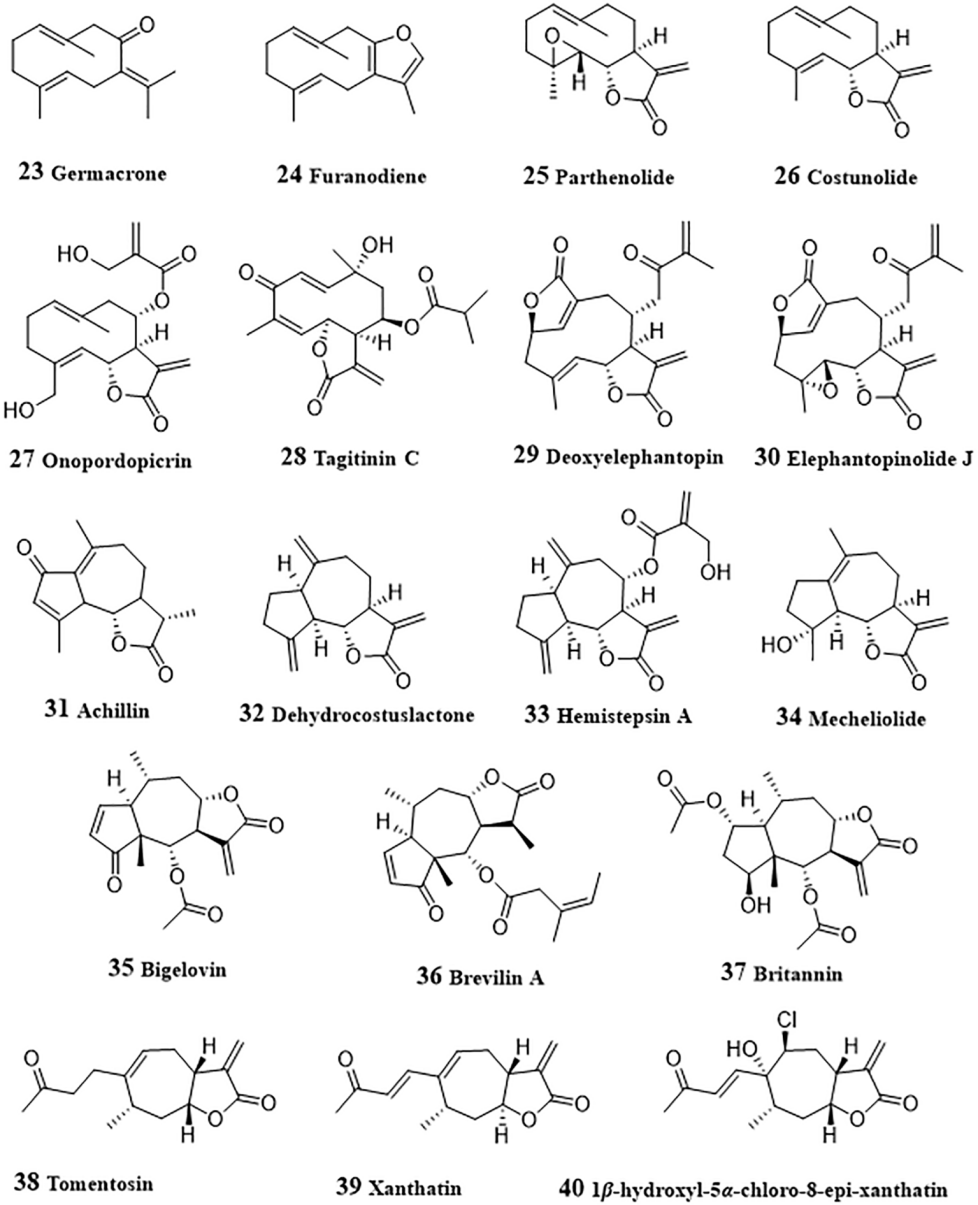
Structures of natural anti-HCC sesquiterpenoids (23 – 40).

Germacrone (**23**, **Figure 3**), a germacrane-type monocyclic sesquiterpenoid derived from Ezhu, a Traditional Chinese medicine from plants of *Curcuma* genus, exhibited anti-tumor effects by inducing apoptosis in a variety of cancers including HCC (125). Germacrone inhibited proliferation both in HepG2 cells and in HepG2 cell xenograft BALB/c nude mice (46). Administration with germacrone could increase the production of ROS, following by promoted cleavage of caspase 3 and gasdermin E, thereby promoting pyroptosis of HepG2 cells. Furanodiene (**24**) is another active sesquiterpenoid from Ezhu and could induce HepG2 cell arrest at G2/M and apoptosis (47). The apoptosis induction effect of furanodiene was achieved by activating mitochondrial signaling pathway, including promoting mitochondrial transmembrane depolarization, release of mitochondrial cytochrome c and cleavage of PARP, and activating caspases−3. Activation of p38, inhibition of ERK, and MAPK signaling were also involved in the apoptosis induced by furanodiene.

Parthenolide (**25**) was a main active sesquiterpene lactone from *Tanacetum parthenium* for treatment of migraines, inflammation, and tumors (126). In invasive sarcomatoid hepatocellular carcinoma cells (SH-J1), depletion of glutathione and ROS generation induced by parthenolide led the overexpression of GADD153 and weakened MMP. With the activation of mitochondrial signaling pathway, caspases-7, -8, and -9 were activated, and following by induction of cell cycle arrest at G2/M phase, thereby triggering apoptosis of SH-J1 cells (48). Parthenolide could also ameliorate the resistance of HepG2, Hep3B, and SK-Hep1 cells to tumor necrosis factor-related apoptosis-inducing ligand (TRAIL)-induced apoptosis (127). The activation of Janus kinase (JAK) was inhibited by parthenolide and resulted in the phosphorylation level of STAT proteins decreased. Decreased STAT induced uptegulation of death receptors TRAIL-R1 and -R2, and apoptosis was facilitated with the activation of caspases-8, and -3. Liu et al. (128) investigated the regulation of parthenolide on metabolic reprogramming of MHCC97-H, a highly-metastatic HCC cells. HIF-1*α*, an important promoter of glycolysis, could induce the cytoplasmic accumulation of pyruvate by the increased pyruvate dehydrogenase activity stimulated by pyruvate dehydrogenase kinase 1, promote glycolysis by up-regulating the glucose transporters (GLUTs). The results by Li et al. indicated that parthenolide targeted NF-κB to inhibit the expression of HIF-1*α*, then inhibit glycolysis and the proliferation of MHCC97-H cells. Due to the potential of parthenolide to be an anti-cancer drug, many structural modification studies have been conducted to improve its druggability (129).

Costunolide (**26**) is a germacrane-type sesquiterpenoid, which exists in many herbal meidicine, such as *Dolomiaea souliei* (Franch.) C. Shih, *Aucklandia lappa* Decne., etc. (130). Costunolide could cause G2/M arrest of cell via mitosis arrest and enhances radiosensitivity of HA22T/VGH HCC cells to ionizing radiation (49). Mitosis arrest induced by costunolide was associated with up-regulated phosphorylation of Chk2, Cdc25c, and Cdk1. Mao et al. (50) investigated the proliferation inhibitory and apoptosis induction effect of costunolide in HepG2 cells. The results showed that, in HepG2 cells, costunolide also arrested cell cycle in G2/M phase in a dose-dependent manner and induced apoptosis by upregulating the expression of Bax, caspases-3, -8 and -9, and downregulating the expression of Bcl-2.

Onopordopicrin (**27**), isolated from *Shangwua* genus could targeting inhibit TrxR through binding of its *β*-carbocation of the lactone ring to the redox-active Sec residue at the C-terminus of TrxR (51). Inhibition of TrxR led to the generation of ROS and induce oxidative stress-mediated tumor cell apoptosis. From *Tithonia diversifolia* leaves, Liao et al. purified a germacrane-type sesquiterpenoid, tagitinin C (**28**), which displayed anti-tumor effect against HepG2 cells and in HepG2 cell xenograft BALB/c nude mice (52).

Deoxyelephantopin (**29**) was found from *Elephantopus scaber*, a traditional Chinese medicinal herb, had been shown the anticancer effects in various cancer cells (131). In HepG2 cells, it could induce apoptosis in a dose-dependent manner by promoting ROS generation, glutathione depletion, loss of MMP, expression of Bcl-2 proteins, cytochrome c release, and PARP cleavage, inhibiting activity of thioredoxin, and activation of caspases-3 and NF-*κ*B (53). Mechanistic study indicated that deoxyelephantopin decreased the phosphorylation of I*κ*B*α* and inhibited the translocation of NF-*κ*B into nucleus, thereby exerting anticancer effects. Deoxyelephantopin could also synergistically induced apoptosis and mitochondrial dysfunction with sorafenib in HepG2 and Hep3B cells, which attributed to its target-binding with Hsp90*α* (132). Some other sesquiterpenoid with anti-HCC activity were also obtained from *Elephantopus scaber*. Elephantopinolide J (**30**) enhanced the ROS production, decrease of MMP, resulting in the autophagy, cell arrest at G2/M phase and apoptosis in HepG2 and Hep3B cells (54). Western blot results suggested that the increased levels of p-p38, p-JNK and p-ERK in MAPKs pathway and reduced p-Akt expression in AKT pathway were associated with apoptosis induced by elephantopinolide J.

### 3.7 Guaiane-type sesquiterpenoids

Achillin (**31**) is a guaianolide-type sesquiterpene lactone obtained from *Artemisia ludovisiana* and acted as a chemosensitizer of paclitaxel in paclitaxel-resistant HCC Hep3B/PTX cells (55). When co-administration of paclitaxel (25 nM) with achillin (100 µM), the induction of G2/M phase cell cycle arrest and apoptosis were promoted. Meanwhile, P-gp levels in Hep3B/PTX cells was decreased and the intracellular retention of doxorubicin was increased by achillin, indicating that achillin exhibited chemosensitization effect through inhibiting drug efflux medicated by P-gp.

Dehydrocostuslactone (**32**) is a guaiane-type sesquiterpenoid, which also exists in *Dolomiaea souliei* (Franch.) C. Shih, *Aucklandia lappa* Decne., and some other herbal meidicine. Treated with 10 mg/kg/day dehydrocostuslactone for 45 days, the growth of PLC/PRF/5 xenografts in nude mice was significantly inhibited with 50% reduction in tumor volume (56). In depth research has found that dehydrocostuslactone could induce HepG2 and PLC/PRF/5 cells apoptosis by up-regulating Bax and Bak, down-regulating Bcl-2 and Bcl-XL, and induce nuclear relocation of the mitochondrial factors AIF and Endo G. Increased cytosol Ca^2+^ level, phosphorylation of PERK and eIF-2α, and protein levels of Bip, IRE1, and CHOP/GADD153 indicated that dehydrocostuslactone could induces ER stress, which contributed to mitochondrial apoptosis. Besides, costunolide and dehydrocostuslactone were also reported to inhibit autophagy in HepG2 cells (133). After treated with costunolide or dehydrocostuslactone, the autophagic flux was blocked, leading to the accumulation of microtubule-associated protein 1 light chain 3 (LC3) and SQSTM1/p62 (p62), thereby resulting in autophagy inhibition. Hemistepsin A (**33**) was isolated from *Hemistepta lyrata* and could induce G0/G1-phase arrest, cellular senescence and mitochondrial-related apoptosis in Huh7 cells and reduce growth of xenografted tumors in mice (57). Hemistepsin A exhibited anti-HCC effect by disrupting MMP, up-regulating p53, p21, cleaved caspase-3 and cleaved PARP and reduced the expressions of cyclin D, CDK6 and Bcl-2. In HepG2 cells, Hemistepsin A-induced apoptosis was associated with its downregulation effect of STAT3 (58). The expression of cleaved PARP cells in Sub-G1 phase were increased, indicating that hemistepsin A induced apoptosis. The decrease of the reduced glutathione/oxidized glutathione ratio and accumulation of reactive oxygen species and glutathione-protein adducts induced by hemistepsin A suggested that oxidative stress participated in hemistepsin A-induced apoptosis. Hemistepsin A also induced dephosphorylation at Y705 of STAT3 and glutathione conjugation, thereby inhibiting STAT3 transactivation. Moreover, hemistepsin A was found to enhance the sensitivity of sorafenib-mediated cytotoxicity in Huh7 cells.

Mecheliolide (**34**), discovered form Aucklandiae Radix, had high potential to induce immunogenic cell death (ICD), a rare immunostimulatory form of cell death, promoted a long-term cancer immunity and significantly stimulated the tumor regression in an immunocompetent mouse vaccine model (59). Mechanically, mecheliolide acted as an inhibitor of TrxR and induced ICD-associated damage-associated molecular patterns (DAMPs, such as CRT exposure, ATP secretion and HMGB1 release). Besides, Mecheliolide-induced ICD was associated with the generation of ROS-mediated endoplasmic reticulum stress (ERS). Suppressing ROS could normalize mecheliolide-induced ERS.

### 3.8 Pseudoguaiane-type sesquiterpenoids

Bigelovin (**35**), isolated from the flower of *Inula hupehensis*, dose-dependently inhibited the IL-6-induced STAT3 activation and induced apoptosis in HepG2 cells transfected with the STAT3-responsive firefly luciferase reporter plasmid (60). Further study showed that JAK2/STAT3 signaling regulating effect of bigelovin was achieved by reacting with cysteine residues of JAK2 leading to inactivation of JAK2. Bigelovin was also found to suppressed the growth of HepG2 cancer xenograft tumors through the activation of apoptosis and autophagy (61). In HepG2 and SMMC-7721 cells, bigelovin promoted the cleavage of Caspase-3 and PARP-1, enhanced the accumulation of autophagosomes, the microtubule-associated light chain 3B-II (LC3B-II) and Beclin-1, and p62 decrease. Meanwhile, bigelovin-induced cell death could be abolished by over-expressing the phosphorylation of mTOR and eliminated by the pretreatment of ROS scavenger NAC, suggesting that ROS played an important role in bigelovin-induced cell death.

Brevilin A (**36**), purified from *Centipeda minima*, was found to reduce cell viability and invasion, induce apoptosis in HepG2 and SMMC-7221 cells (62). The expressions of matrix metalloproteinase-2, matrix metalloproteinase-9, p-STAT3, STAT3, Snail, *β*-catenin, and *c*-Myc decreased after the treatment with brevilin A, indicating the inactivation of STAT3/Snail and Wnt/*β*-catenin signaling pathways was involved in the anti-HCC activity of brevilin A.

Britannin (**37**), isolated from *Inula aucheriana*, significantly suppressed the growth of Huh-7, HepG2 and SMMC-7221 cells through the extrinsic and intrinsic apoptotic pathways (63). Mechanistic study revealed that britannin increased the cleavages of Caspase-8, -9 and -3, triggered autophagy by the up-regulating LC3 II, p62, autophagy-related 5 (ATG5) and Beclin 1, and induced ROS generation. Moreover, AMPK activation was increased while the activity of mTOR decreased. All these results revealed that britannin induce apoptosis and autophagy by activating AMPK regulated by ROS. Britanin also inhibited p65 protein expression and reduced the ratio of Bcl-2/Bax, suggesting its antitumor effect by regulation of NF-κB signaling pathway (64).

### 3.9 Xanthane-type sesquiterpenoids

Tomentosin (**38**), a sesquiterpene lactone, induced DNA fragmentation, cell cycle arrest at the SubG1 and G2/M stage and apoptosis in HepG2 and Huh7 cells (65). Treated with tomentosin, the expressions of Bax, Bim, cleaved PARP1, FOXO3, p53, pSer15p53, pSer20p53, pSer46p53, p21, and p27 increased, with expressions of Bcl2, caspase3, caspase7, caspase9, cyclin-dependent kinase 2 (CDK2), CDK4, CDK6, cyclinB1, cyclinD1, cyclinD2, cyclinD3, and cyclinE decreased in HepG2 and Huh7 cells. Xanthatin (**39**), a bicyclic sesquiterpene lactone from *Xanthium strumarium*, could arrest cell cycle at the G2/M phase at 10 μM, and at the S phase at 40 μM in HepG2, SMMC-7721 and Bel-7402 cells (66). The activated PERK/eIF-2α/ATF4 axis by promoting nuclear translocation of ATF4 and increased the of CHOP levels and cleaved-caspase-3 indicated that xanthatin induced apoptosis of HCC cells by activating ERS. A xanthatin analogue 1*β*-hydroxyl-5*α*-chloro-8-epi-xanthatin (**40**), isolated from *Xanthium sibiricum*, inhibited the cell growth and induced apoptosis in HepG2, SMMC-7721, Huh7 and SK-Hep-1 cells via induction of Bax and cleaved-caspase-3, inhibition of Bcl-2 and survivin expression (67). ROS-mediated ERK/p38 MAPK activation and JAK2/STAT3 inhibition were involved in the apoptosis, including induction of generation of ROS and malondialdehyde (MDA), GSH depletion, activation of extracellular regulated protein kinase (ERK) and p38 mitogen-activated protein kinase (p38 MAPK) and inactivation of JAK2/STAT3.

### 3.10 Sesquiterpenoid dimers

**Figure 4:**
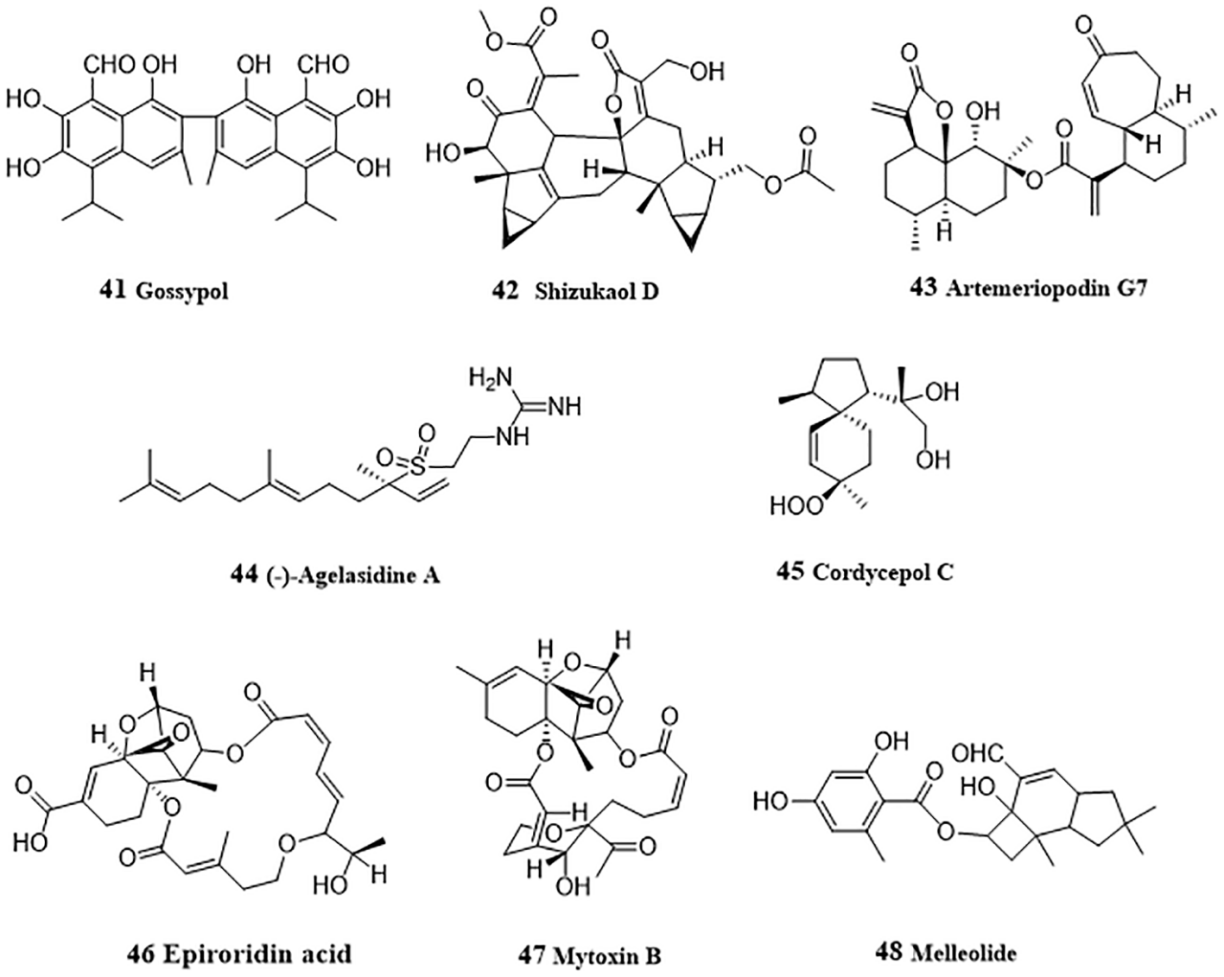
Structures of natural anti-HCC sesquiterpenoids (41 – 48).

Gossypol (**41**, **Figure 4**), a sesquiterpenoid dimer existed in cotton seeds, acted as a pan-histone deacetylase (HADC) inhibitor and inhibited cellular viability and proliferation in HepG2 and Hep3B cells (68). Gossypol induced hyperacetylation of histone protein H3 and/or tubulin and inhibited HDAC histone deacetylase activity. In-depth mechanism study showed that gossypol induced apoptosis via caspase activation. Shizukaol D (**42**), a sesquiterpenoid dimer from *Chloranthus serratus*, induced apoptosis in SMMC-7721, SK-HEP1 and HepG2 cells by repressing Wnt/β-catenin signaling (69). Shizukaol D reduced the expression of endogenous Wnt target genes, including decreased expression of β-catenin, down regulation of Disheveled 2 (Dvl2) and Axin2, reduction of phosphorylation of co-receptor LRP6, and repressed C-Myc, Cyclin D, Tcf-1, LEF1, wnt3a and FGF18. Artemeriopodin G7 (**43**) was obtained from *Artemisia eriopoda*, and displayed anti-hepatoma effect by inhibiting AKT/STAT signaling pathway (70). In HepG2 cells, artemeriopodin G7 induced cell cycle arrest in G2/M phase and apoptosis by downregulation of the expressions of phosphorylated cdc2 and AKT/STAT, Bcl-2, upregulation of the expression of CyclinB1, Bax, and PDGFRA. PDGFRA may be its directly target, which was evidenced via cellular thermal shift assay, isothermal titration calorimetry, and surface plasmon resonance assay.

### 3.11 Other sesquiterpenoids

(-)-Agelasidine A (**44**), a sesquiterpene guanidine obtained from sea sponge, *Agelas nakamurai*, induced apoptosis in Hep3B cells (71). Reduced MMP and cytochrome c release after treatment with (-)-agelasidine A indicated the activation of mitochondrial signaling pathway. Activation of caspases 9, 8, 3, and PARP, together with the increased apoptosis-associated proteins (DR4, DR5, FAS, Bim, and Bax), and decreased Bcl-2 protein promoted by (-)-agelasidine A indicated apoptosis. Moreover, ER stress-related proteins (GRP78, phosphorylated PERK, phosphorylated eIF2α, ATF4, truncated ATF6, and CHOP) were also upregulated by (-)-agelasidine A in Hep3B cells. Cordycepol C (**45**) was separated from the cultured mycelia of Cordyceps ophioglossoides and induced HepG2 cells caspase-independent apoptosis by causing PARP-1 cleavage and MMP loss in a time- and dose-dependent manner, leading the nuclear translocation of AIF and Endo G, and up-regulated expression and translocation from cytosol to mitochondria of Bax (72). From the endophytic fungus *Myrothecium roridum* derived from *Pogostemon cablin*, two trichothecene toxins, epiroridin acid (**46**) and mytoxin B (**47**) were isolated and displayed apoptosis-inductive effect in HepG2 cells (73). Mechanically, these two mycotoxins activated caspase-9 and caspase-3, up-regulated Bax level, down-regulated bcl-2 level, and reduced MMP, thereby exhibiting anti-HCC activity. From *Armillaria mellea* (Vahl. ex. Fr.) Karst., Li et al. (74) purified an anti-HCC protoilludane sesquiterpene aryl ester, melleolide (**48**). In HepG2 cells, melleolide increased the expressions of cleaved caspase 3, caspase 8, caspase 9, Bax and Ki67, and induced G2/M phase cell cycle arrest.

## 4 Discussion and conclusion

Natural products have always been an important source of new drug development (9). The properties of high safety, synergy and little side-effects *in vivo* of sesquiterpenoids make them promising anti-liver cancer treatments. For example, dihydroartemisinin and melleolide had a relative minor cytotoxicity toward normal cell lines (18, 74). Artesunate, *β*-caryophyllene, *β*-Caryophyllene oxide synergized with sorafenib (25, 30). *β*-Caryophyllene oxide synergized with doxorubicin (33). Though few compounds have actually entered clinical research, many efforts have been made in the new drug development of sesquiterpenoids for the treatment of liver cancer. The studies on the pharmacokinetic characteristics, tolerability of artesunate in colorectal cancer patients, stage I breast cancer, and metastatic breast cancer patients were conducted. Dose dependent toxicity of artesunate was found in patients with metastatic breast cancer. The maximum tolerated dose of intravenous artesunate was 18 mg/kg in patients with advanced solid malignant tumor (16). Biomimetic nanomedicine of dihydroartemisinin with high drug loading efficiency showed better anti-HCC activity (21).

Sesquiterpenoids are frequently liposoluble with bad bioavailability. Many of them had the disadvantage of narrow therapeutic window or poor bioavailability. Synthetic chemists have carried out variety of investigations by structural modification or designing completely new compounds to improve their druggability. Structure reform of active sesquiterpenoids to improve their water-solubilities and anticancer efficacies provide many potential anti-HCC candidates (35, 129). A ludartin derivative,1,4-bis(3*α*-hydroxy-4*β*-hydroxy-5,7*α*,6*β*(*H*)-guaia-1 (10),11 (13)-dien-12,6-olide)phthalate displayed cytotoxic effect about 20 times stronger than ludartin (IC_50_: HepG2 1.6 vs 32.7 μM and Huh7 2.0 vs 34.3 μM) (134). To solve the problem of poor water-solubility of parthenolide, Taleghani et al. (135) prepared the novel parthenolide derivatives, parthabine and parthalan, which displayed better water-solubility and stronger cytotoxic effect against HepG2 cells.

Many researchers are also devoted to discover new anti-HCC sesquiterpenoids from natural materials. A variety of new discovered sesquiterpenoids were found to possess anti-HCC effect. Wang et al. (136) found that the essential oil of *Backhousia citriodora* could induces HepG2 cells S-phase cell cycle arrest and apoptosis. From *Vernonia extensa*, three elemane-type sesquiterpenoids, vernodalin, vernolepin, and vernolide were purified and exhibited apoptosis-inductive effect in HepG2 cells (137). Guaiane-type sesquiterpenoid dimers from *Artemisia atrovirens* also exhibited significant inhibition effect on cell migration and invasion, induced G2/M cell cycle arrest and cell apoptosis in HepG2 cells (138).

Mitochondria, as the central organelles that maintain cell growth and apoptosis, regulate apoptosis-related signaling pathways. So mitochondrial apoptosis pathway has become an important pathway for treating cancer. The studies on the apoptosis-inductive mechanism of several sesquiterpenoids has been conducted. For instance, artesunate was found to target STAT-3 and favorably suppress HCC (23). Parthenoilide inhibited HIF-1*α*-mediated metabolic reprogramming of HCC by targeting NF-κB (128). Onopordopicrin targeted TrxR to induce oxidative stress-mediated apoptosis (51). Besides, sesquiterpenoids could also exert anti-HCC effect by regulating some other biological process, such as inducing cell cycle arrest, inhibiting invasion and metastasis (1), inducing autophagy (96), serving as chemosensitizer (25, 30, 33), etc. The complex mechanism of anti-tumor activities of many sesquiterpenoids are still unclear. Further researches are needed to reveal the targets of these active sesquiterpenoids.

Sesquiterpenoids are abundant in nature and have great potential for the development of new anti-HCC drugs. As a result, this review summarized the anti-HCC sesquiterpenoids and their mechanism of action. We believe that more studies in the future on the pharmacological effect, therapeutic mechanism, structural modification, formulation development of sesquiterpenoids will provide more contributions for the discovery and development of novel anti-HCC therapies.

## Author contributions

XW: Conceptualization, Methodology, Software, Visualization, Writing – original draft. FM: Formal analysis, Visualization, Writing – original draft. JM: Conceptualization, Funding acquisition, Methodology, Project administration, Supervision, Writing – review & editing.
